# Supplementary figures and images for: G2Φnet: Relating genotype and biomechanical phenotype of tissues with deep learning
Source: PLoS Comput Biol. 2022 Oct 31;18(10):e1010660. doi: 10.1371/journal.pcbi.1010660 (PMC9668200; doi:10.1371/journal.pcbi.1010660)

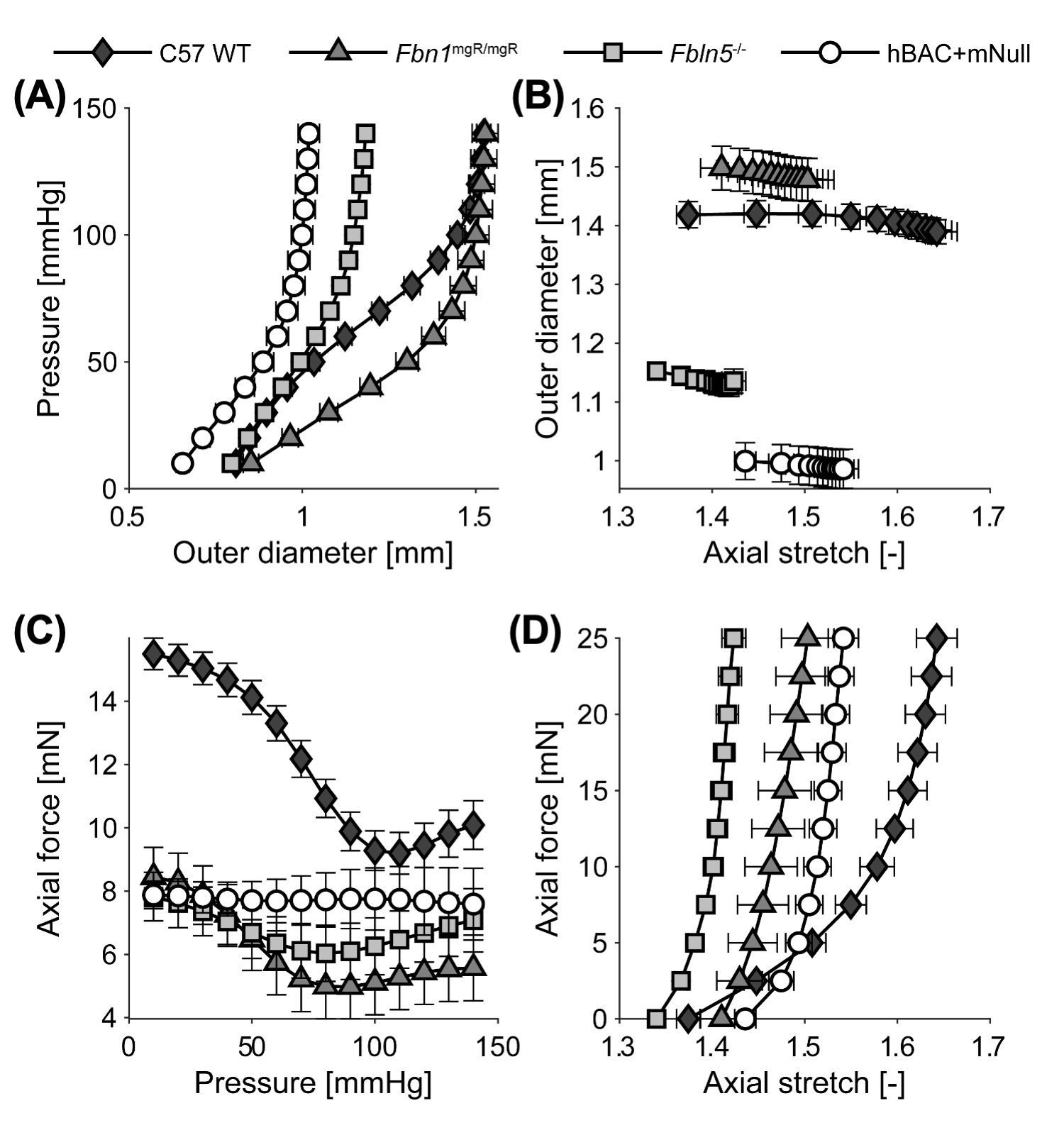

Supplement: S1 Fig — Data were obtained through biaxial tests using a seven-step protocol, including three inflation cycles at axial stretch (λz) levels of 95%, 100%, and 105% of the in vivo axial stretch (λz,iv) and four extension cycles at pressures (P) of 10, 60, 100, and 140 mmHg (C and D). Whiskers indicate standard error (n = 8, 8, 5, 7 for the four genotypes from left to right of the legend). Data are shown for the same single (1 of 7) protocol for each of the genotypes. (TIF) [file pcbi.1010660.s001.tif]

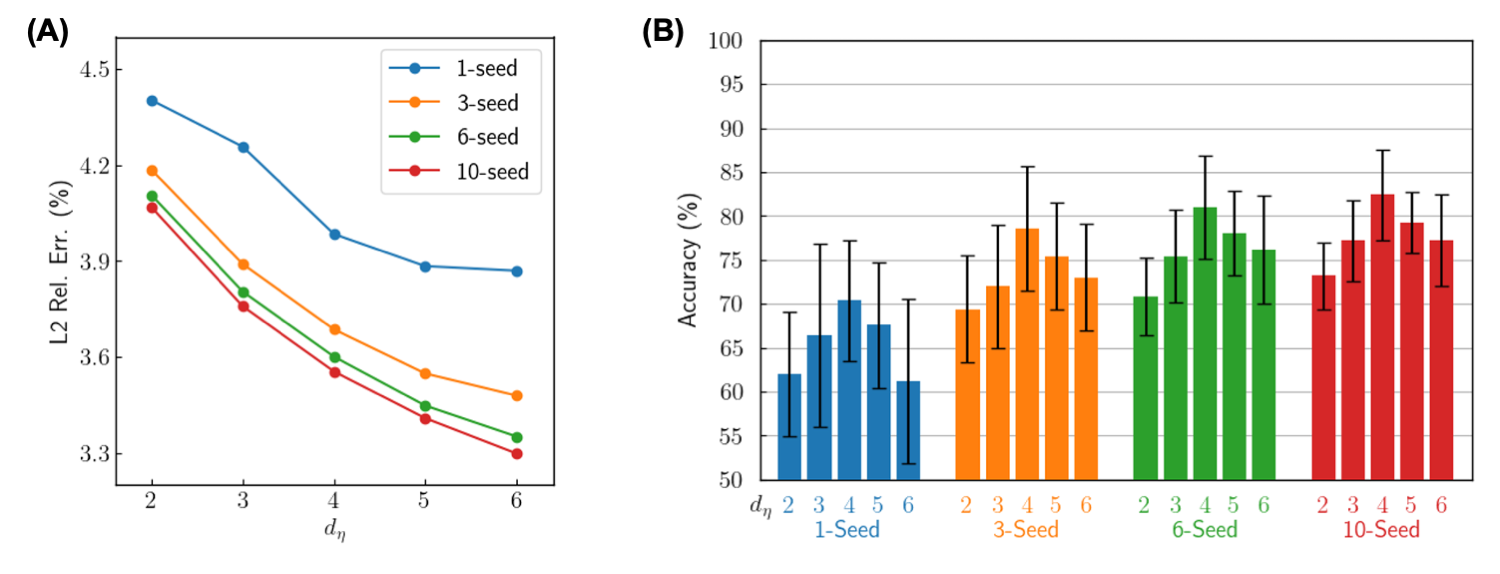

Supplement: S2 Fig — Samples with five genotypes, where two of them (WT and hBAC-mWT) share similar microstructure and hence exhibit similar mechanical properties, are involved. (A) L2 relative (Rel.) error of the predicted normalized stress σ˜i with different sizes of ensemble (1-, 3-, 6- and 10-seed) and different dimensions of sample feature (dη ∈ {2, 3, 4, 5, 6}). Each dot is an average value over 20 runs with different random seeds. (B) Classification accuracy for different sizes of ensemble and different dimensions of sample feature. Each bar is the average value over 20 runs with different random seeds. (TIF) [file pcbi.1010660.s002.tif]

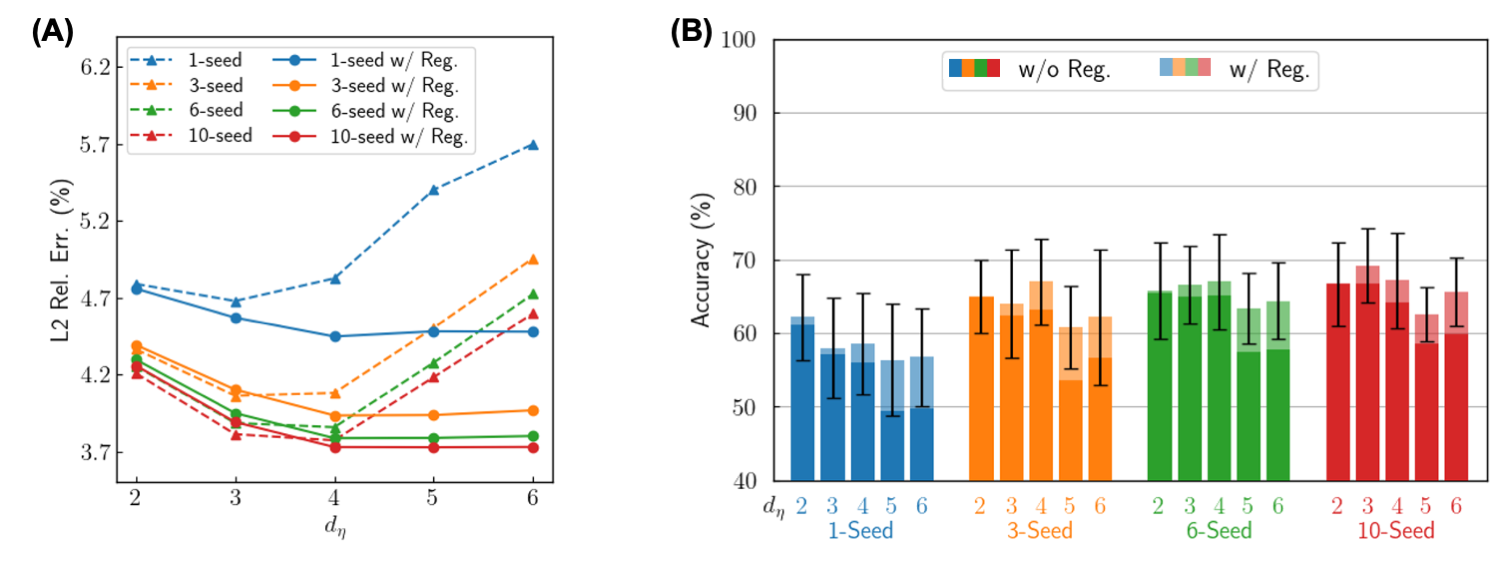

Supplement: S3 Fig — Samples with five genotypes, where two of them (WT and hBAC-mWT) share similar microstructure and hence exhibit similar mechanical properties, are involved. (A) L2 relative (Rel.) error of the predicted normalized stress σ˜i with different sizes of ensemble (1-, 3-, 6- and 10-seed), different dimensions (dη ∈ {2, 3, 4, 5, 6}) and regularization (Reg.) for the sample feature (without/with regularization). Each dot is the average value over 20 runs with different random seeds. (B) Classification accuracy for different sizes of ensemble, different dimensions and different regularization setups of sample feature. Each bar is the average value over 20 runs with different random seeds. (TIF) [file pcbi.1010660.s003.tif]
