# Supplementary material for: G2Φnet: Relating genotype and biomechanical phenotype of tissues with deep learning
Source: PLoS Comput Biol. 2022 Oct 31;18(10):e1010660. doi: 10.1371/journal.pcbi.1010660 (PMC9668200; doi:10.1371/journal.pcbi.1010660)
Supplement: S1 Appendix — (PDF) [file pcbi.1010660.s004.pdf]

S1 Appendix. Technical Details of G2Φnet

G2Φnet processes stress data by their log-transformed value defined in Eq. 1. To further ensure that the neural network deals with stress data that roughly locate within $[-1, 1]$, the log-transformed value of stress is divided by 5.0 before feeding into G2Φnet.

BE consists of four convolutional layers each with 64 output channels, with kernel sizes 5, 3, 3, 3 and strides 2, 2, 1, 1, respectively. The output after convolutional layers is then flattened and fed into three dense layers (with sizes $[256, 64, 16, d_{\boldsymbol{\eta}}]$). BD consists of four dense layers (with sizes $[d_{\boldsymbol{\zeta}} + d_{\boldsymbol{\eta}}, 48, 48, 48, 2p]$ where $p = 128$). TN consists of four dense layers (with sizes $[2, 48, 48, 48, 2p]$ where $p = 128$). Among the $2p$ components in the outputs of BD and TN, p components are used to calculate $\hat{\sigma}_\theta$ and $\hat{\sigma}_z$, respectively (see Eqs. 5-8). BE and BD use the ReLU activation function for all layers except the final layers (no activation). Note that TN needs to use the Tanh activation function for all layers to ensure that the stress-stretch relationship is smooth.

In the learning stage, the learning rate is initially 0.001 and halves every 5000 epochs. We set $\varepsilon = 0.02$, $w_{\text{rec}} = 1.0$, $w_{\text{reg}} = 0.01$; $v_k = 0.01$ if the kth data is synthetic and $v_k = 1$ otherwise. To balance the four genotypes with imbalanced dataset (8, 8, 5, and 7 samples, respectively), we oversample certain data points from the minority classes (i.e., the genotype with 5 and 7 samples) to make sure that the data is balanced. We generate 56 synthetic samples by mixup. Both the mixup and oversampling are random and changes every epoch. The number of copies saved in the training process is $K_2 = 3$, corresponding to 16K, 18K, and 20K epochs.

In the inference stage, the learning rate is 0.1. We set $\varepsilon = 0.02$, $w_{\text{fit}} = 1.0$, and $w_{\text{reg2}} = 10^{-5}$ (for Setup 2 with raw unstructured data only). Sample feature $\boldsymbol{\eta}$ is defined as trainable variable and directly fed into BD. To define the class feature, we firstly define logits $\boldsymbol{\zeta}' \in \mathbb{R}^{d_{\boldsymbol{\zeta}}}$, and then feed $\boldsymbol{\zeta}'$ into a softmax layer to calculate the class feature $\boldsymbol{\zeta} = \text{Softmax}(\boldsymbol{\zeta}')$. In this way, we ensure that the class feature $\boldsymbol{\zeta}$ is nonnegative and sums to 1.

We conduct five-fold cross-validation in our study. Specifically, as we have 8, 8, 5 and 7 samples for the four genotypes, we keep one sample from every genotype as test sample each time, resulting in 24 training samples and 4 testing samples.
